# Fibrosis, Adipogenesis, and Muscle Atrophy in Congenital Muscular Torticollis

**DOI:** 10.1097/MD.0000000000000138

**Published:** 2014-11-14

**Authors:** Huan-xiong Chen, Sheng-ping Tang, Fu-tang Gao, Jiang-Long Xu, Xian-ping Jiang, Juan Cao, Gui-bing Fu, Ke Sun, Shi-zhe Liu, Wei Shi

**Affiliations:** From the Zunyi Medical College Zhuhai Campus, Zhuhai (H-XC); Department of Pediatric Orthopaedics (S-PT, F-TG, J-LX, G-BF, KS, S-ZL, WS); and Department of Pediatric Pathology, Shenzhen Children's Hospital, Shenzhen, China (X-PJ, JC).

## Abstract

In the traditional view, muscle atrophy and interstitial fibrosis were regarded as the basic pathological features of congenital muscular torticollis (CMT). But in the ultrastructure study, the mesenchyme-like cells, myoblasts, myofibroblasts, and fibroblasts were found in the proliferation of interstitium of CMT. To investigate the characteristics of pathological features and the mechanisms of muscle atrophy in CMT, we retrospectively reviewed the medical records of 185 CMT patients from July 2009 to July 2011 in Shenzhen Children's Hospital in China and performed pathological studies. According to age, the 185 CMT patients were divided into 4 groups. All resected surgical specimens were processed for hematoxylin and eosin staining and Masson trichromic staining. Sudan III staining was used for frozen sections, whereas immunohistochemical staining for S-100, calpain-1, ubiquitin, and 20S proteasome was carried out on 40 CMT specimens. Eight adductor muscle specimens from 8 patients with development dysplasia of the hip were taken as control group in the immunohistochemical staining. By Masson trichromic staining, the differences in the percent area of fibrous tissue in each CMT groups were significant. In Sudan III staining and immunostaining for S-100, adipocyte hyperplasia was the pathological feature of CMT. Moreover, compared with controls, most atrophic muscle fibers in CMT specimens were found to show strong immunoreactivity for calpain-1, ubiquitin, and 20S proteasome. With increasing age, fibrosis peaked at both sides and it was low in middle age group. Adipocytes increased with age. **T**he characteristics of pathological features in CMT are changeable with age. The calpain and the ubiquitin–proteasome system may play a role in muscle atrophy of CMT. In the CMT, adipogenesis, fibrogenesis, and myogenesis may be the results of mesenchyme-like cells in SCM (sternocleidomastoid muscle). In conclusion, the present study furthermore supports maldevelopment of the fetal SCM theory for etiology of CMT.

## INTRODUCTION

Although congenital muscular torticollis (CMT) was known for centuries, its etiology is still a topic of debate today.^1,2^ Histologically, collagen and fibroblasts were observed around atrophic muscle fibers.^3^ In 1998, Tang et al^4^ reported the ultrastructure of the sternocleidomatoid muscle (SCM) tumor of infants with CMT. In the interstitium, there were not only collagen but also fibroblasts, mesenchyme-like cells, myoblasts, and myofibroblasts. In fetal muscle, a large number of mesenchymal stem cells (MSCs) exist. Although the vast majority of MSCs commit to myogenesis, they are also capable of differentiating into other cell types, such as adipocytes or fibroblasts.^5^ Theoretically, if the pathogenesis of CMT was related to embryogenesis of SCM, then muscle, fibrous, and adipose tissue may also exist in the SCM of CMT. In addition, if the physiological functions of skeletal muscle were impaired by the adipose tissue or fibrous tissue, skeletal muscle may undergo atrophy by the calpain and the ubiquitin–proteasome system.^6–8^ However, to our knowledge, no research reported adipose tissue hyperplasia and nor the mechanism of muscle atrophy in CMT. Therefore, we used a histological and immunohistochemical analysis to detect fibrous tissue, adipose tissue, and the mechanism of muscle atrophy in CMT so as to find out more pathological characteristics in SCM of CMT.

## METHODS

### Patients

Between July 2009 and July 2011, a total of 185 CMT patients were operated at the Department of Pediatric Orthopaedics of Shenzhen Children's Hospital, China. This study retrospectively reviewed the patients’ clinical feature and analyzed pathological findings. Among the 185 patients with CMT, 62 girls and 123 boys were present, the mean age was 34.6 months (range 3 months–16 years) at the time of the operation and the incidence of breech presentation was 27.56%. Fifty CMT patients were born via cesarean section and none of these patients had a history of birth trauma. All patients with CMT were diagnosed by clinical manifestations and ultrasonography of the SCM. The surgical operation indications for CMT patients, younger than 1 year, were patients with significant craniofacial asymmetry, severe deformational plagiocephaly, and poor response to 3 months of manual stretching physiotherapy. According to children's growth, development, and age at operation, the 185 CMT patients were divided into 4 groups: group 1, 33 patients (3–6 months); group 2, 55 patients (7–12 months); group 3, 49 patients (1–3 years); group 4, 48 patients (4–16 years). Eight adductor muscle specimens from 8 patients from same hospital (mean age 2.1 years) with development dysplasia of the hip were taken as control group for histologic and immunohistochemical staining. The inclusion criteria for control specimens were normal or only minimal histological changes in hematoxylin and eosin (H&E) staining.^9^ All specimens were harvested during operation and fixed immediately in 10% formaldehyde solution and embedded in paraffin after routine tissue processing. Moreover, the proximal half of 31 CMT specimens was additionally processed for frozen sections. Informed consent was obtained from parents of all the patients. The study was approved by the Ethics Committee of Shenzhen Children's Hospital.

### Histochemistry

Serial paraffin sections of 4 mm for each specimen were prepared and stained with H&E and Masson trichromic stain for routine histolopathological evaluation. Moreover, for detecting intramyocellular fat droplets and intramuscular fat, that is, fat globules between muscle ﬁbers, the unstained frozen sections was using Sudan III staining, in which fat (triglyceride) was stained in a distinct yellow orange.

In Masson trichromic staining, each slide was examined under Leica microscope (Leica, Wetzlar, Germany) and analyzed with Image-Pro Plus software (Media Cybernetics, Silver Spring, USA). Under ×100 magniﬁcation, 5 images were taken randomly in each slide. The percent area of fibrous tissue was then expressed as the percent ratio of fibrous tissue area to the area of the both fibrous tissue and muscle ﬁbers in each image. In each slide, the 5 images were selected for calculating the mean percent area of fibrous tissue.

### Immunocytochemistry

To distinguish intramuscular adipocytes from the vacuolated defect due to the apoptosis of muscle fibers in CMT by H&E staining, 40 CMT specimens were used immunostaining for S-100. Moreover, according to an age-stratified and random way, 40 specimens were collected from 185 patients with CMT (ages 4 months–16 years) for immunohistochemical staining for calpain-1, ubiquitin, and 20S proteasome.

Tissue samples were paraffin-embedded and cut into serial sections of 4 μm in thickness, followed by dewaxing with xylene and dehydrating in a series of graded ethanol. For antigen retrieval, sections were immersed in 10 mmol/L sodium citrate buffer, pH 6.0 (Maxim, Fujian, China), autoclaved at 120°C for 3 minutes, and cooled to room temperature. Intrinsic peroxide activity was blocked by immersion in distilled water containing 3% hydrogen peroxide for 10 minutes. Each section was incubated for 10 minutes in phosphate-buffered saline (PBS) containing 10% (w/v) normal goat serum. Unstained serial sections were incubated overnight at 4°C with a monoclonal antibody for S-100 (diluted 1:100; Maxim, Fujian, China) and polyclonal antibodies for calpain-1 (diluted 1:200; Boster, Wuhan, China), for ubiquitin (diluted 1:100; Maxim), and for 20S proteasomes (diluted 1:200; ProteinTech, Wuhan, China). After 3 washes in PBS, the Elivision peroxidase kit and DAB chromogen (Maxim) were used following the manufacturer's recommendations. All slides were double-stained with hematoxylin for demonstrating the cell nucleus. Control sections were treated with PBS instead of the specific antibody. Staining was photographed with a Leica microscope and was analyzed with Image-Pro Plus software (Media Cybernetics, Silver Spring, USA).

After H&E staining and immunostaining for S-100, under ×100 magniﬁcation, 5 images were taken randomly in each slide. The amount of adipocytes on the 5 images was calculated. The amount of adipocytes was graded with a 5-scale system (0, 1, 2, 3, or 4), where a grade 0 = no adipocyte deposits, grade 1 = 1–10 (the amount of adipocytes), grade 2 = 10–20, grade 3 = 20–50, and grade 4 ≥ 50. Quantitative analysis with immunostaining for calpain-1, ubiquitin, and 20S proteasome was applied to CMT group and control group. Under ×400 magnification, 10 images were taken randomly in each slide. The percentage of strongly calpain-1-, ubiquitin-, and 20S proteasome-positive muscle fibers to all muscle number was counted in each slide.

### Statistical Analysis

For the percent area of fibrous tissue, groups were compared using one-way analysis of variance. The correlation between age and the amount of intramuscular adipocytes was analyzed by Spearman rank correlation coefficients. For the percentage of strongly calpain-1-, ubiquitin-, and 20S proteasome-positive muscle fibers, differences between CMT group and control group were evaluated with an unpaired *t* test. *P* < 0.05 was considered statistically significant.

## RESULTS

### Histochemistry

**FIGURE 1:**
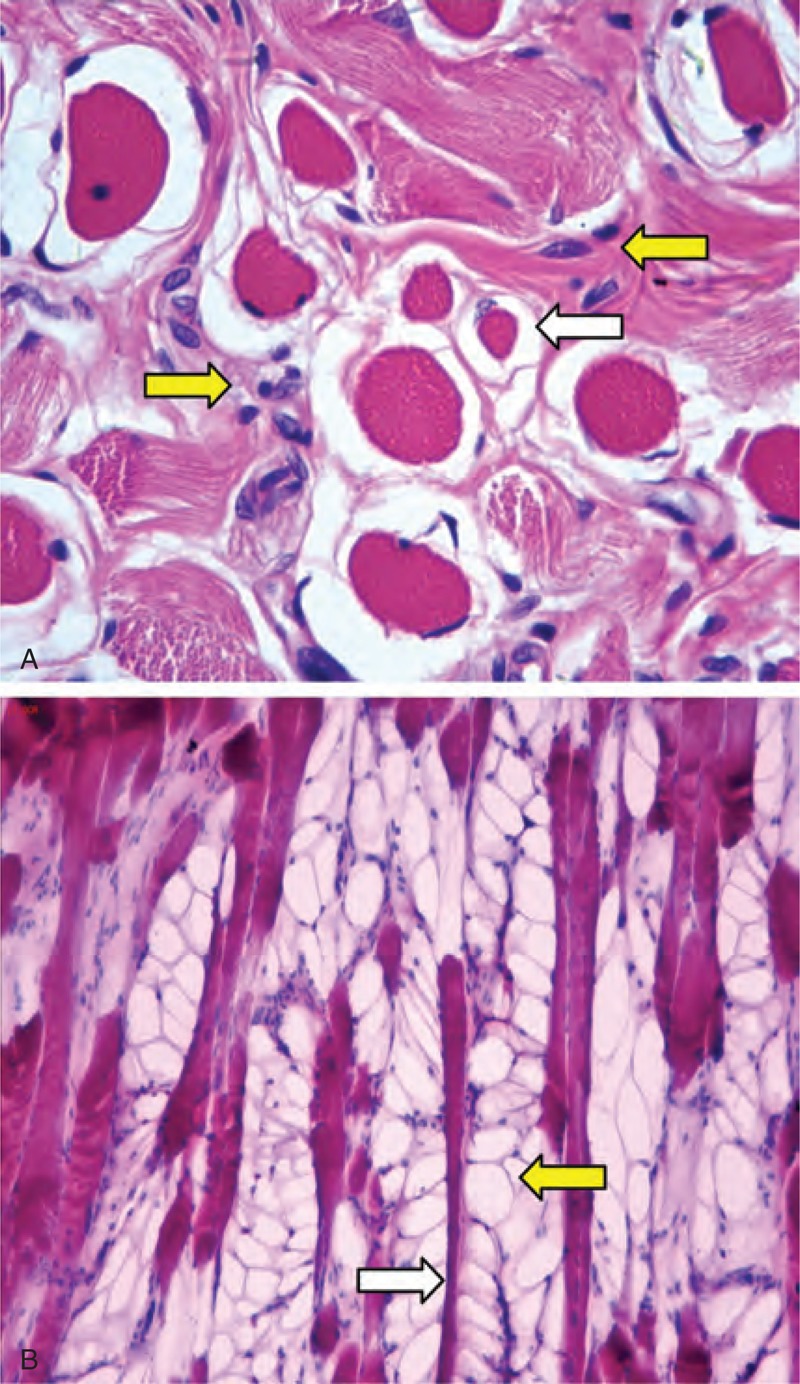
A–B Histological micrographs of a sample of a 12-month-old patient with CMT show (A) the atrophic muscle fibers (white arrow) surrounded by the proliferating fibroblasts (yellow arrow) (H&E staining, ×400 original magniﬁcation). In a sample of a 17-month-old CMT patient, longitudinal section shows (B) the adipocyte hyperplasia (yellow arrow) around atrophic muscle fibers (white arrow) (H&E staining, original magniﬁcation ×100).

**FIGURE 2:**
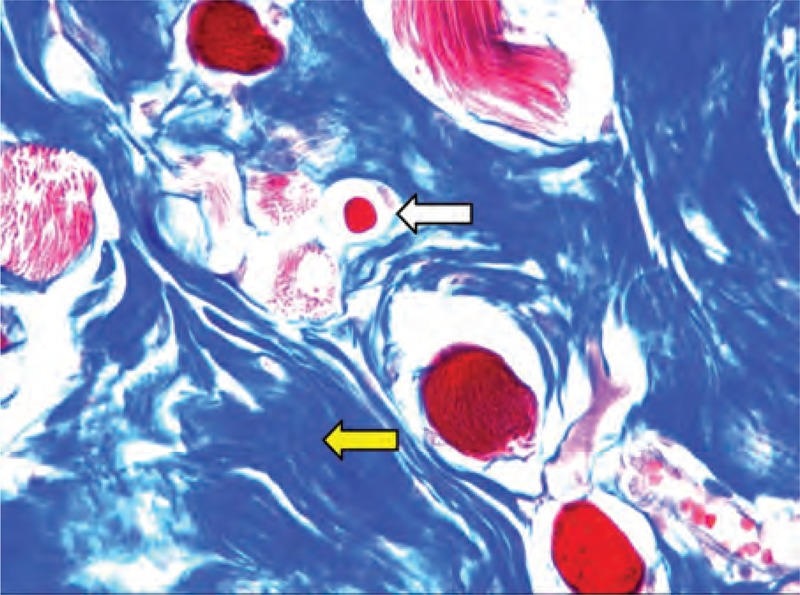
Histological micrographs of a sample of a 12-month-old CMT patient show the blue proliferating collagen (yellow arrow) around atrophic muscle fibers (white arrow) (Masson trichromic staining, ×400 original magniﬁcation).

**FIGURE 3:**
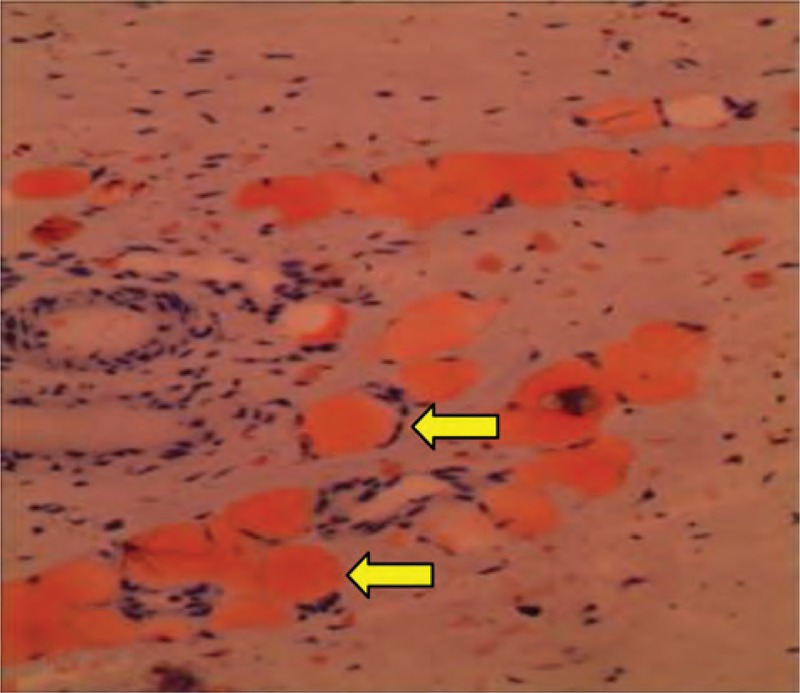
The frozen section of a 3-year-old CMT patient shows positive staining for adipocytes (yellow arrow) (Sudan III staining, ×200 original magniﬁcation).

In H&E staining, according to the observation of 185 CMT specimens, the pathological features of CMT included excessively perimysial and endomysial fibrosis, adipocyte hyperplasia, and muscle atrophy (Figure 1). By Masson trichromic staining, atrophic muscle fibers divided by the excessively proliferating collagen in varying degrees were observed in all 185 CMT specimens (Figure 2). In Sudan III staining, the frozen sections of CMT specimens showed positive staining for adipocyte (Figure 3). However, no intramyocellular fat droplet was found in these CMT specimens. According to the result of histological studies, other abnormalities in CMT also included vacuolar degeneration of muscle fibers, centralized myonuclei, and multinucleated muscle giant cells. But, none of 185 CMT specimens showed inflammatory cellular infiltration, cell necrosis, and hemosiderin deposition.

In Masson trichromic staining, the differences in the percent area of fibrous tissue in CMT groups were significant and the average of percent area of fibrous tissue was 55% in the group 1, 48% in the group 2, 46% in the group 3, and 55% in the group 4, respectively (Table 1).

**TABLE 1:**
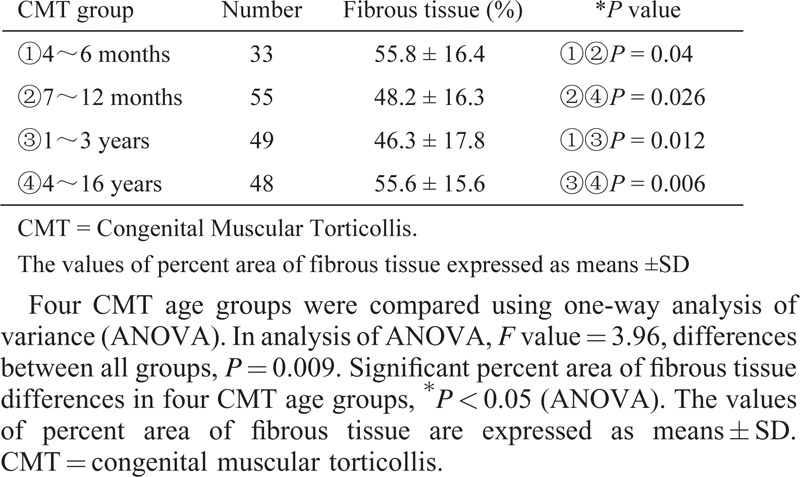
Percent Area of Fibrous Tissue Examined in 4 CMT Age Groups

### Immunohistochemistry

#### S-100

**FIGURE 4:**
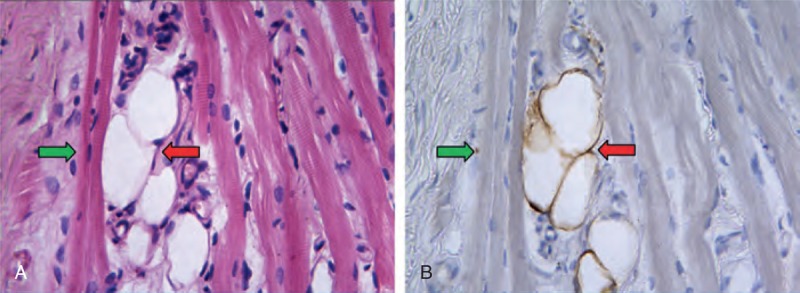
A–B By serial sections, (A) H&E staining and (B) immunostaining for S-100 showed adipocytes (red arrow) surrounded by muscle ﬁbers (green arrow) (×400 Original magniﬁcation).

In CMT specimens, immunohistochemical reaction for S-l00 protein was positive in nervous tissue and adipose tissue where the reaction product was located in both the nucleus and the narrow cytoplasm. In control group, histological and immunostaining for S-100 showed that only a few of adipocytes scattered in perimysium. Compared with controls, adipocyte proliferation was observed in perimysium and endomysium of the 154 CMT specimens. By serial sections, H&E staining and immunostaining for S-100 showed adipocytes surrounded by muscle fibers (Figure 4).

By H&E staining and immunostaining for S-100, the differences in the amount of adipocytes in CMT groups were significant and the average of grade was 1.84 in the group 1, 1.98 in the group 2, 2.28 in the group 3, and 2.85 in the group 4, respectively. In the CMT groups, the amount of adipocytes was positively correlated with age (*r* = 0.257, *P* = <0.01).

#### Calpain-1, Ubiquitin, and 20S Proteasome

**FIGURE 5:**
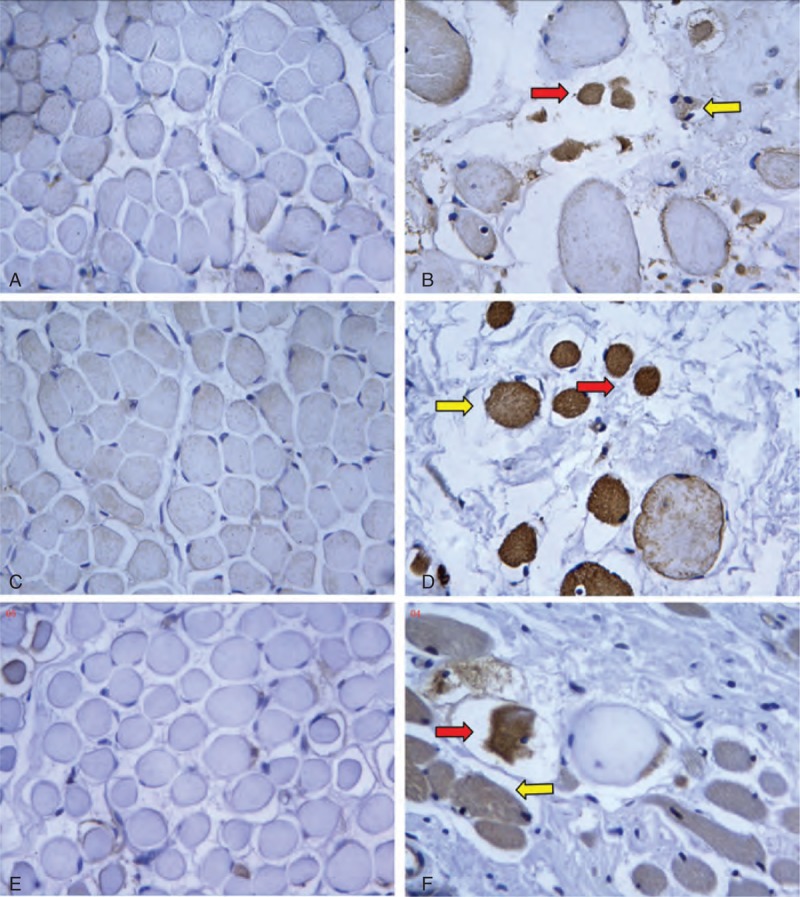
The sections obtained from muscles of controls (13 months’ old) and 2-year-old patients with CMT. Immunostaining for (A) calpain-1, (C) cubiquitin, and (E) 20S proteasome were very weak in the muscle cytoplasm of the control. But, in the CMT specimen, atrophic muscle fibers showed moderate (yellow arrow) or strong (red arrow) immunoreactions for (B) calpain-1, (D) ubiquitin, and (F) 20S proteasome (×400 original magniﬁcation).

The sarcoplasm of muscles in the control group exhibited only a faint immunohistochemical reaction for calpain-1, ubiquitin, and 20S proteasome. Compared with muscles in the control group, the muscles in CMT demonstrated remarkable positive stain by calpain-1, ubiquitin, and 20S proteasome, which were expressed mainly in the cytoplasm of atrophic muscle fibers (Figure 5). The percentage of strongly calpain-1-, ubiquitin-, and 20S proteasome-positive fibers in CMT group was significantly higher than controls (P < 0.01) (Table 2).

**TABLE 2:**
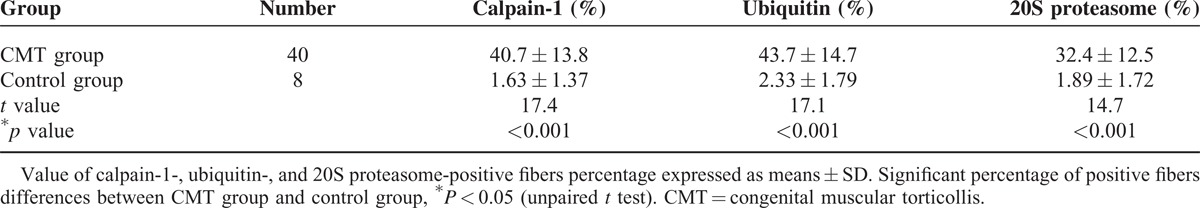
Percentage of Positive Fibers in CMT Group and Control Group

## DISCUSSION

The etiology of CMT is still a topic of debate. Multiple theories exist, including birth trauma, compartment syndrome, and maldevelopment of the fetal SCM.^4,10–13^ It was believed that, in 1875, Taylor^3^ was the first researcher to describe main pathological symptom of sternocleidomastoid tumor. Since then, the fibrosis in CMT was regarded as the basic pathological characteristic. However, the mass in SCM has capability to increase in size and diminish spontaneously by several months and with different clinic outcomes. It also has complex and changeable sonographic appearance under the follow-up.^14^ The clinic feature and ultrasonography of CMT cannot be explained by SCM fibrosis. The finding of the ultrastructure research in CMT indicates that there are numerous mesenchyme-like cells, myoblasts, myofibrobalsts, and fibroblasts in the interstium of CMT.^4^ The mesenchyme-like cell is capable of differentiating into muscle, fibrous, and adipose tissues. As a result, the basic pathology of CMT should include fibrogenesis, adipogenesis, and myogenesis or muscle atrophy in addition to fibrosis. To best of our knowledge based on the published English literature, no adipocyte was found out in the pathological tissue of CMT.

Fetal stage is crucial for skeletal muscle development because there is no net increase in the number of muscle fiber after birth.^5^ Furthermore, the late fetal stage is also very important for adipogenesis, which forms intramuscular adipocytes.^8^ In fetal muscle, a large number of MSCs exist. Although the vast majority of MSCs commit to myogenesis, they are also capable of differentiating into other cell types, such as adipocytes or fibroblasts. A shift from myogenesis to adipogenesis or fibrogenesis will replace muscle fibers with adipose or fibrous tissues.^5,15,16^ By previous ultrastructure research, the CMT specimens showed that mesenchyme-like cells existed in proliferating interstitium.^4^ In the present research, H&E staining, Sudan III staining, and immunostaining for S-100 showed that intramuscular adipocyte hyperplasia is the pathological feature of CMT. Besides, in the 185 CMT specimens, the amount of adipocytes increased with age. These results suggested that fibrogenesis, adipogenesis, and myogenesis may result from MSC differentiation in the SCM of CMT after birth.

Fibrosis and adipogenesis originate from a common mesenchymal progenitor in skeletal muscle.^15,16^ However, the proliferation of both fibrous and adipose tissue will impair the physiological functions of skeletal muscle.^5^ In this research, most atrophic muscle fibers in CMT were found to show strong subsarcolemmal immunoreactivity for calpain-1-, ubiquitin-, and 20S proteasome. Moreover, the same atrophic muscle fiber showed strong immunoreactions for the 3 proteins. These results indicate that the proliferation of both fibrous and adipose tissue in CMT lead to muscle atrophy by the calpain and the ubiquitin–proteasome system.

According to the observation of both clinical and pathological features of 185 CMT patients, this research demonstrated that 50 CMT patients were born via cesarean section, none of these patients had a history of birth trauma, and none of 185 CMT specimens showed inflammatory cellular infiltration, cell necrosis, and hemosiderin deposition. Therefore, the results of this research were inconsistent with birth trauma theory and the compartment syndrome theory.

On the progression of the SCM mass in CMT, if the mass disappeared and lead to fibrosis of SCM after 1 year of age, the SCM would be hardened and contracture, the CMT patients required subsequent surgical treatment.^17^ The fibrous tissue proliferation is basic abnormality of the SCM mass in CMT.^4,18^ In this research, by Masson trichromic staining, within 1 year of age, the percent area of fibrous tissue in 3- to 6-month age group was more than 7- to 12-month age group, whereas, after 1 year of age, the percent area of fibrous tissue in 1- to 3-year age group was less than 4- to 16-year age group. These findings were remarkably similar to the progression of the contracture of SCM in CMT. Moreover, this study showed, with increasing age, the percent area of fibrous tissue peaked at both sides and it was low in middle age group (1–3 years). Base on this finding, it could infer that conservative treatment should be applied for CMT patients with 3–6 months old even though the mass is harder and the surgical procedure should be better methods for the patient elder than one year of age if the SCM contracture is insisted on.

In our previous ultrastructure study, the mesenchyme-like cells and myoblasts were regarded as key points to CMT pathologic characteristics.^4^ In the present research, adipogenesis, fibrogenesis, and myogenesis may be the results of mesenchyme-like cells in SCM. In conclusion, by connecting the clinic feature of CMT to the previous ultrastructure research and by histological and immunohistochemical analysis of specimens for CMT in this study, the present study furthermore supports maldevelopment of the fetal SCM theory as etiology of CMT.
